# Sleep Pattern Changes in Nursing Students during the COVID-19 Lockdown

**DOI:** 10.3390/ijerph17145222

**Published:** 2020-07-20

**Authors:** Cristina Romero-Blanco, Julián Rodríguez-Almagro, María Dolores Onieva-Zafra, María Laura Parra-Fernández, María del Carmen Prado-Laguna, Antonio Hernández-Martínez

**Affiliations:** Department of Nursing, Physiotherapy and Occupational Therapy, Ciudad Real Faculty of Nursing, University of Castilla-La Mancha, 13071 Ciudad Real, Spain; Cristina.romero@uclm.es (C.R.-B.); mariadolores.onieva@uclm.es (M.D.O.-Z.); marialaura.parra@uclm.es (M.L.P.-F.); carmina.prado@uclm.es (M.d.C.P.-L.); antonio.hmartinez@uclm.es (A.H.-M.)

**Keywords:** COVID-19, nursing, lockdown, sleep, sleep disorders

## Abstract

The prevalence of poor sleep quality among students is very high and, in nursing students, has been associated with reduced performance, behavioral changes, dietary changes, and even aggressive behavior due to changes in sleep patterns. The lockdown in response to COVID-19 may have resulted in lifestyle changes that affected sleep quality. For this reason, the objective of this study is to determine the difference in nursing students’ sleep quality before and during the lockdown, put in place in response to the coronavirus (COVID-19) pandemic. To meet this objective, we conducted a longitudinal observational study on 207 nursing students, with two cut-off points (February and April). The main dependent variable was sleep quality, measured using the Pittsburgh sleep quality index (PSQI) and its seven components. Parametric and nonparametric tests were used for paired and unpaired data, as well as group-stratified analysis. The mean time students spent in bed was 7.6 h (standard deviation (SD) = 1.1 h) before lockdown and 8.5 h (SD = 1.2 h) during lockdown. The PSQI score got 0.91 points worse during lockdown (95% CI, −0.51, −1.31). Of the five components, five were statistically significantly affected (*p* ≤ 0.05), and of these, the most changed were sleep latency, sleep duration, and sleep efficiency. When stratified by group, we observed differences in women, first-year students, second-year students, alcohol consumers, those of normal weight, and those that live with family. The main conclusion is that although students spent more time in bed, overall sleep quality was worse during lockdown, as well as being worse in five of the seven components.

## 1. Introduction

Coronavirus disease (COVID-19) has been declared an international emergency by the World Health Organization (WHO). The rapid spread of the disease has meant that unprecedented restrictions have been implemented to control its spread and mitigate its impact [1].

In response to the outbreak, the Spanish government issued a royal decree (463/2020) declaring a 15-day national emergency, closing the country's borders on 15 March [2].

These measures were accompanied by a series of social distancing measures, including closing schools, universities, retirement homes, and sports venues, and restricting all movement in the most affected areas [2]. 

During previous outbreaks, the psychological impact on the noninfected population revealed significant psychological morbidities, negative emotions, and sleep problems [3]. An existing study in patients with SARS (severe acute respiratory syndrome) reported that people in quarantine were more likely to have symptoms of insomnia [4].

The current COVID-19 pandemic is causing psychosocial problems such as stress, worry, fear, anxiety, depressive symptoms, and sleep disorders in the general public [5,6,7,8]. It is also resulting in low levels of sleep quality caused by stress and anxiety [9].

University students may be one of the populations affected. In recent months, university students have modified their routines due to the COVID-19 pandemic. We also need to consider that the transition from secondary education to university already involves significant changes for students, with sleep being one of the most common problems they face [10]. On top of the usual changes that altered sleep patterns cause, the lockdown has forced teaching systems to adapt, allowing students to adapt their study hours since they no longer have to attend their usual morning classes. All of these changes to teaching and schedules are an additional factor in sleep problems, as well as affecting academic performance [11,12]. Although few studies have reported on stress levels caused by the lockdown in Spain, one recent study [13] showed that stress levels increased in the Spanish population after the lockdown was imposed and that those most affected by stress, anxiety, and depression were those aged 18–25.

The prevalence of sleep disorders is quite high among the general population and is associated with significant morbidity and mortality [14]. It is estimated that among university students, the prevalence of poor sleep quality is around 60% [15] or even higher. 

Previous studies talk about impaired performance, behavioral changes, dietary changes, and even aggression in nursing students caused by altered sleep patterns [16,17,18]. 

The effects of lockdown on sleep disorders are not yet very well understood, but we know that the population spent more time in bed, spent more time on digital devices close to bedtime, went to bed and got up later, and their sleep quality worsened [19].

Our aim was to identify whether university students would experience changes in sleep quality during lockdown compared to normal times. The purpose of this study was, therefore, to analyze sleep quality and its various associated factors during face-to-face teaching (before the pandemic) and compare it with that observed during remote teaching (during the pandemic).

The hypothesis of this study was that students' sleep quality worsened due to the pandemic and the resulting lockdown.

## 2. Material and Methods

### 2.1. Design and Selection of Study Subjects

A longitudinal study was carried out on nursing students, with two sample points. The first sample point was between 15 to 30 January, 2020, prior to the state of alarm being put in place, and the second sample point was between 1 and 15 April, 2020. This study has received the approval of the Ethics and Clinical Research Committee of Ciudad Real, Spain, with a protocol number (C-291, 11/2019).

This study was carried out within the context of another study that we conducted on healthy habits and lifestyles, with an estimated follow-up period of 9 months. Due to the state of alarm and lockdown, recruitment of subjects was suspended, so we decided to study the impact of lockdown on the population already participating. There were no exclusion criteria, other than failure to fully complete the questionnaire.

To estimate the sample considering a bilateral hypothesis, the following criteria were used: variance in the pre-lockdown control group of 8.35, obtained using the Pittsburgh sleep quality index, a beta risk of 20% (power = 80%), a confidence level of 95%, and a clinically important difference of 0.8 points on the Pittsburgh scale in overall sleep pattern alteration with respect to the control group. It was therefore estimated that 205 study subjects would be needed.

We used an ad hoc self-administered questionnaire and collected sociodemographic information such as sex, age, weight, height, place of residence during the academic year, smoking habits, and alcohol consumption. Tobacco and alcohol consumption were dichotomized as yes/no. For perceived health status, the EQ-5D questionnaire (Euro quality of life - 5 dimensions) was used [20]. 

To assess adherence to the Mediterranean diet, we used the PREvencion con DIeta MEDiterranea (PREDIMED) questionnaire [21], which uses 14 questions to assess the frequency of food consumption and eating habits. Each question had a possible score of 0 or 1. The results allow classification into low adherence or high adherence. 

We also asked participants how many minutes of moderate and intense physical activity they did a week, to assess whether they met the physical activity recommendations of the World Health Organization (WHO) [22]. 

The main dependent variable was sleep quality, measured using the Pittsburgh sleep quality index (PSQI) [23]. The PSQI contains 19 items and seven clinically important components in relation to sleep difficulties: subjective sleep quality, sleep latency, sleep duration, sleep efficiency, sleep disturbance, use of sleep medication, and daytime dysfunction. Responses were given based on a Likert-type scale from 0 to 4. To evaluate them, a sleep profile was obtained for each of the components, ranging between 0 and 3, as well a global score of between 0 and 21. Global sleep scores of 5 or less were deemed good quality; scores above 5 were deemed low quality.

The survey was conducted during the second university semester. The first data collection point was two weeks after exams, while the second data collection point was 4 weeks after the start of the lockdown. Students in their first three years of study attended between 5 and 8 h of theory classes a day (morning and evening schedule), starting at 9 in the morning, while final-year students attended clinical practice from Monday to Friday for 7 h a day (morning shift), starting at 8 in the morning. 

At the second data collection point, the students had not left their homes in one month; theory classes were being held virtually. The schedule for the virtual classes was the same as before lockdown. In Spain, it was forbidden to leave home except for essential purposes such as buying food or going to the hospital. Those who violated the rules were fined 600 euros or even imprisoned. 

### 2.2. Statistical Analysis Used

First, we performed descriptive statistics using absolute and relative frequencies for categorical variables and mean with standard deviation (SD) for the quantitative variables. Next, we performed bivariate analysis on the whole sample for paired data between the global PSQI scores and its components for the two sample points (pre-lockdown and lockdown). For the analysis, we used the parametric Student-Fisher *t*-test and the nonparametric Wilcoxon signed-rank test.

Finally, we performed the same analyses again, but this time stratified for different subgroups. We obtained mean differences (MD) with a confidence interval of 95% (CI). Next, we analyzed the relationship between different sociodemographic and lifestyle factors and sleep quality (PSQI). To do this, we used repeated measures analyses of covariance (ANCOVA) to estimate the mean difference in PSQI scores and the eta squared (η^2^) effect size. For the comparisons, we used the Bonferroni method.

All calculations were done using the program SPSS v24.0 (IBM Corp, New York, NY, USA).

## 3. Results

A total of 207 nursing students participated in this study, after excluding ten students with incomplete responses. The mean age was 20.6 years (SD = 4.62; age range 17–53). 81.6% of the participants (169) were women, 75.8% (157) were of normal weight, and 9.7% (20) were smokers. The rest of the demographic characteristics and health parameters are shown in Table 1.

Next, we studied the sleep changes that occurred between the two sample points, observing statistically significant differences overall (global PSQI score), in sleep efficiency, and in five of the seven components that make up the PSQI, both in the parametric and nonparametric tests. The three most changed components were, in descending order: sleep latency, sleep duration, and sleep efficiency. The PSQI score got 0.91 points worse during lockdown (95% CI, −0.51, −1.31). Further information is shown in Table 2. 

The prevalence of poor sleep quality in the sample analyzed was 60.4% at the first sample point and 67.1% at the second.

The times at which the students went to bed and got up were assessed at both points in time, and we observed statistically significant differences (*p* < 0.001) for both of these parameters. The students’ usual bedtime was delayed by slightly more than one hour (1.08 ± 1.2), and the time the students got up was two hours later than it was during face-to-face teaching (2.0 ± 1.4). 

The mean time the students spent in bed was 7.6 h (SD = 1.1 h) before lockdown and 8.5 h (SD = 1.2 h) during lockdown. 

Next, we stratified the sample by group and analyzed the variation between the PSQI scores obtained at both time periods (Table 3). We observed statistically significant differences, with lower PSQI scores during lockdown with respect to the before lockdown in the following categories: women, normal weight, first-year students, second-year students, alcohol drinkers, and living with parents. We also observed statistically significant differences between the two periods, but these differences were present in all categories that made up the strata smoking habit, anxiety/depression, adherence to a Mediterranean diet, and physical activity recommendations.

Finally, when we did the repeated measures ANCOVA, we observed that only smoking and anxiety/depression were related to factors that could influence sleep quality. Specifically, smokers’ experienced a reduction in sleep quality of 2.29 points (95% CI, 0.73–3.85) with respect to nonsmokers, and students that reported anxiety/depression experienced a reduction in sleep quality of 1.74 points (95% CI, 0.85–2.63) with respect to those that did not.

## 4. Discussion

The effects of COVID-19 go beyond physical health. It also has psychological consequences including emotional distress, anxiety, fear, depression, suicidal tendencies, public stigma, discrimination, racism, xenophobia, post-traumatic symptoms, and sleep disorders [24].

We found differences in the students’ sleep quality between both periods analyzed, with worse sleep quality during the lockdown. In the stratified analysis, we observed that this worsening of sleep quality was maintained in the subgroups women, first-year students, and second-year students. We also observed that these differences remain statistically significant in the quality of sleep before and during lockdown for those with a normal BMI, those who consumed alcohol, and those who lived with family during the academic year. Although worse quality of sleep was seen in other subgroups, these differences were not statistically significant due to their low statistical power, as these subgroups had a much smaller sample size. 

In relation to the data obtained in this study, recent research suggests that lockdown has worsened sleep quality despite increasing its quantity, observing that there has been an increase in mental health problems such as anxiety, depression, and even suicidal thoughts [18,25]. It seems that the pandemic and the fear of infection have increased suicidal ideation, with insomnia having a significant impact: the more severe the insomnia, the greater the effect on these types of thoughts [26]. Therefore, this study’s stratification of sleep quality before and after lockdown could be very useful for future interventions.

The Pittsburgh index (PSQI) has been used to assess sleep quality in adults for many years [27]. In our results, the students obtained scores above five in the pre-lockdown stage and scores above six in the post-lockdown stage, in line with recent studies that reported PSQI scores higher than 5 in 85% of subjects, which is considered pathological [28].

Looking at the results obtained at each of the time points analyzed, we observed that at the first data collection point, the PSQI score was around 5 (the borderline sleep quality score) in all students except smokers and those with anxiety or depression, who had higher scores. Conversely, nondrinkers and those with a low BMI obtained the lowest scores.

At the second data collection point, smokers obtained average scores of 8.9, followed by those with anxiety or depression. The lowest scores at the second data collection point were again nondrinkers and students with a lower BMI. Although the relationship between BMI and sleep quality is not very clear, it seems that an inverse relationship between these two parameters had already been observed in a previous study [29]. In the general population, there is a close relationship between obesity and sleep problems, with unhealthy eating habits and inadequate physical activity also having an influence [30,31]. In this study, the PSQI score obtained at each of the two time points analyzed was higher at higher BMIs. However, we only observed changes in sleep quality among students with a normal weight, perhaps because of the low sample sizes in the other categories.

Regarding tobacco and alcohol consumption, poor sleep quality is related to alcohol consumption [32] and smoking [33] in university students, as observed in previous studies. Both drinkers and smokers showed significant increases in their PSQI scores during lockdown, but, as we have already mentioned, smokers achieved the worst sleep quality scores out of all the groups analyzed. Poor sleep quality is associated with smokers, although this data is not always easy to observe in self-reported questionnaires and more specific data are needed [34]. In this case, when assessing the effect on smokers and on nonsmokers, we found significantly worse sleep quality in both groups during lockdown. However, while nonsmokers scored around six on the PSQI, smokers scored higher than eight, on average. 

The criteria for poor sleep quality differ depending on gender [35], with gender differences in variables such as latency and waking during the night, and women experiencing more problems than men [36]. In this study, both genders obtained similar total scores; however, lockdown caused significant changes in women but not in men.

In all the sleep parameters analyzed, higher scores were obtained during lockdown, indicating worse sleep quality, except in component 3 (duration of sleep). Students slept more hours, as we can see from the score in this component and the differences in the times the participants went to bed and the times they got up. On the surface, this might suggest better quality sleep, but in fact, the opposite is true. At both the sample points, sleep duration (component 3) received a healthy score, as both values exceeded seven hours. Despite an increase in the number of hours spent in bed, component 4 shows that sleep efficiency (the ratio between time in bed and actual sleep time) declined during the lockdown. In other words, even though students spent more hours in bed, they took longer to fall asleep.

Although the PSQI global scores increased, indicating worsening of sleep quality, sleep timing delayed (indicative of potentially lower social jetlag due to a discrepancy between endogenous circadian rhythm and actual sleep times imposed by social obligations). This might be favorable or beneficial to students since they no longer need to wake up early for class [37], but the effects of lockdown were more detrimental to students' sleep, and the sleep timing delay did not strongly affect.

Of the 7 components analyzed, we observed the biggest differences in the second, sleep latency. Sleep latency refers to the time it took students to fall asleep, with subjects asked how often they had trouble getting to sleep within 30 minutes. In university students, this parameter is related to internet addiction [38]. These data, together with recent studies showing that the population increased their use of technology before going to bed during lockdown [18], explain our findings.

As well as analyzing each of the components, we looked at the sleep efficiency score. This parameter is evaluated in component 4. Although there is a certain amount of controversy about which values should be considered normal and even how efficiency should be measured [39], the optimum PSQI score of 0 is for those with a habitual sleep efficiency above 85%. In this study, mean values remained around this figure at both time points analyzed, but we did observe significant differences, with mean values of 86.19% during lockdown compared to 89.57% before lockdown. 

We found no significant differences in the component “use of sleep medication”. Some authors think that component 6 may not be advisable in measuring the overall sleep quality score in young adults [23], although this parameter is undoubtedly very interesting when it comes to establishing the prognosis and treatment for sleep problems.

Similarly, we observed no differences in component 7, daytime dysfunction, which refers to sleepiness while performing everyday activities. Previous studies looking at daytime dysfunction in university students [40] observed that it worsened as the academic year went on, although not significantly. Based on the results we obtained, it seems that this component and component 6 are those least affected by lockdown.

In the Spanish population, poor sleep quality has been linked to poor health, poor diet, and low physical activity [41]. In this study, the PSQI scores were similar in both sets of data; lockdown significantly worsened the participants’ sleep quality regardless of whether they adhered to the Mediterranean diet. In terms of physical activity, this study assessed whether compliance with the 150 minutes of physical activity per week had any bearing on the global PSQI score. The results showed significant changes, although the scores were similar for both groups. We did not collect any data on the type of physical activity (aerobic/anaerobic) carried out by participants, nor did we examine sleep efficiency or other parameters in relation to physical activity. Previous studies have observed that physical activity had a beneficial effect on sleep quality, although it did not affect all components equally, and there were also differences depending on the type of exercise the subjects did [42]. 

In their review, Dinis et al. observed a correlation between sleep quality and depression [43]. Other factors, like stress, also affect university students’ sleep patterns [15]. In this study, lockdown worsens sleep quality both for those who reported problems with anxiety or depression and those who did not. However, those that reported anxiety or depression had initial sleep quality scores one point higher than those who did not and, as the repeated measures ANCOVA shows, anxiety/depression and smoking were presented as factors that could influence the change in sleep quality.

Regarding the students’ year of study, the initial sleep quality scores were similar, although they were lower according to the earlier the year of study. First- and second-year students experienced significant changes during lockdown. The initial years in nursing studies are mainly theoretical, becoming much more practical as time goes on, with a greater number of clinical credits. Previous studies on medical students in their final years of study and with clinical contact found high levels of stress and poor sleep quality [44]. This could also be the case for nursing students. Also, the fact that students in earlier years of study experienced significantly worse sleep quality during lockdown suggests that the same reasoning applies; students in their final years of study had their placements canceled, while students in their first years of study had to adapt to a new way of teaching and virtual assessments in order to pass the year, which could have increased stress and worsened sleep quality. 

Another interesting aspect was the place of residence during the academic year. Before lockdown, scores are similar for each variable, however, lockdown only caused significant changes to those living with family. Living with other students during the academic year, such as at university residences or shared flats, had an influence on sleep quality [45]. All students living in university residences or rented flats had to return to their family homes and this could have improved sleep quality, preventing any significant differences in the scores during lockdown.

### Strengths and Limitations

Our study has various limitations that should be considered. Firstly, it is an observational study, all study subjects volunteered to participate in the questionnaire and there were no exclusion criteria, so there may be a selection bias. Secondly, we did not measure whether there was any risk of exposure to COVID-19 infection, a factor that could have influenced sleep quality in the study subjects. Another limitation is the reliance on self-reported sleep measures as opposed to objective measures (for example, actigraphy). Finally, the lack of significance in some of the strata analyzed may be due to the lack of statistical power.

As for the strengths, this is the first study that compares sleep disturbance just before and during the lockdown in Spain.

## 5. Conclusions

Although students spent more time in bed during lockdown, we observed a reduction in sleep quality, in terms of both global PSQI score and in five of its components, during the COVID-19 lockdown. 

The sleep parameters related to the use of medication and daytime dysfunction saw no changes.

We did not observe any relationship between sleep quality during lockdown and physical activity, eating habits, tobacco consumption, or anxiety/depression. Conversely, gender, body mass index, year of study, alcohol consumption, and place of residence during the academic year seem to be related to sleep quality and the effects of lockdown.

More complete studies should be carried out that include accelerometry data and evaluate the long-term impact of these changes. It would also be interesting to analyze the effect of late chronotype and its relationship to poor sleep quality among students.

## Figures and Tables

**Table 1:** Descriptive characteristics of the population.

	% (*N*)	Mean (SD)
**Age**		20.57 (4.62)
**Gender**		
Male	18.4 (38)	
Female	81.6 (169)	
**Year of study**		
First year	40.1 (83)	
Second year	28.0 (58)	
Third year	21.3 (44)	
Fourth year	10.6 (22)	
**BMI**		22.12 (3.56)
Normal	75.8 (157)	
Underweight	12.6 (26)	
Overweight or Obese	11.6 (24)	
**Alcohol Consumption**		2.23 (1.64) ^a^
Yes	82.6 (171)	
No	17.4 (36)	
**Smoker**		0.68 (2.49) ^b^
Yes	9.7 (20)	
No	90.3 (187)	
**Health status (scale 0–100)**		78.68 (15.13)
**Anxiety/Depression**		
Yes	23.7 (49)	
No	76.3 (158)	
**Adherence to Mediterranean diet**		7.65 (1.81) ^c^
Yes	30.9 (64)	
No	69.1 (143)	
**Follows physical activity recommendations**		221.80 (306.96) ^d^
Yes	44.0 (91)	
No	56.0 (116)	
**Living situation**		
University residence	18.8 (39)	
Shared apartment	42.5 (88)	
With family	33.4 (69)	
Other	5.3 (11)	

SD: Standard deviation. ^a^ drinks alcohol every time they go out; ^b^ cigarettes per day; ^c^ PREDIMED score; ^d^ minutes of physical activity per week.

**Table 2:** Changes in the sleep components analyzed during lockdown. Parametric and nonparametric bivariate analysis.

	Pre (*N* = 207)		Post (*N* = 207)		MD	CI 95%		*p* *t*-Student	*p* Wilcoxon
	Mean	SD	Mean	SD					
**COMP 1** **Subjective sleep quality**	1.03	0.71	1.26	0.84	−0.227	−0.332	−0.122	<0.001 *	<0.001 *
**COMP 2** **Sleep latency**	1.03	0.89	1.54	1.06	−0.515	−0.653	−0.376	<0.001 *	<0.001 *
**COMP 3** **Sleep duration**	0.87	0.73	0.57	0.71	0.295	0.180	0.410	<0.001 *	<0.001 *
**COMP 4** **Sleep efficiency**	0.42	0.76	0.65	0.89	−0.232	−0.364	−0.100	0.001 *	0.001 *
**COMP 5** **Sleep disturbance**	1.03	0.39	1.12	0.43	−0.082	−0.149	−0.015	0.017 *	0.017 *
**COMP 6** **Use of sleep medication**	0.14	0.49	0.16	0.54	−0.029	−0.108	0.050	0.468	0.450
**COMP 7** **Daytime dysfunction**	0.99	0.78	1.11	0.88	−0.121	−0.248	0.007	0.063	0.068
**PSQI Global score**	5.51	2.89	6.42	3.36	−0.908	−1.307	−0.509	<0.001 *	<0.001 *
**Percentage sleep efficiency**	89.57	11.14	86.19	12.47	3.37	1.49	5.25	<0.001 *	0.001 *
**Mean bedtime**	00:42	1.05	01:50	1.42	−1.08	−1.25	−0.92	<0.001 *	<0.001 *
**Mean get-up time**	08:01	0.93	10:01	1.42	−2.00	−1.81	−2.19	<0.001 *	<0.001 *
**Mean time spent in bed**	7.59	1.10	8.51	1.15	−0.92	−0.75	−1.09	<0.001 *	<0.001 *

MD, mean difference; SD, standard deviation; * Statistically significant differences.

**Table 3:** PSQI score pre- and post-stratification by group. Parametric and nonparametric bivariate analysis.

	Pre	Post	MD	95% CI		*p* *t*-Student	*p* Wilcoxon	Repeated Measures ANCOVA			
	(Mean ± SD)	(Mean ± SD)						MD (I–J)	95% CI	*p* Value	*η^2^* Effect Size
**Gender**											0.003
Male	5.53 ± 2.95	5.97 ± 3.69	−0.45	−1.45	0.55	0.371	0.868	Ref			
Female	5.50 ± 2.89	6.51 ± 3.29	−1.01	−1.45	−0.57	**<0.001**	**<0.001**	–0.40	–1.40, 0.60	0.430	
**Year of study**											0.011
First year	5.40 ± 2.66	6,36 ± 3.15	–0.96	–1.64	–0.29	**0.006**	**0.013**	**Ref**			
Second year	5.28 ± 2.91	6.22 ± 3.38	–0.95	–1.70	–0.20	**0.014**	**0.024**	0.15	–1.07, 1.36	1.000	
Third year	5.82 ± 3.19	6.59 ± 3.43	–0.77	–1.62	0.08	0.074	0.123	–0.44	–1.78, 0.90	1.000	
Fourth year	5.91 ± 3.16	6.77 ± 4.13	–0.86	–2.06	0.33	0.148	0.147	–0.63	–2.34, 1.08	1.000	
**BMI**											0.022
Normal	5.59 ± 2.98	6.52 ± 3.30	–0.93	–1.36	–0.50	**<0.001**	**<0.001**	**Ref**			
Underweight	4.85 ± 2.11	5.35 ± 2.83	–0.50	–1.79	0.79	0.434	0.612	1.19	–0.19, 2.57	0.115	
Overweight or Obese	5.71 ± 3.00	6.92 ± 4.15	–1.21	–2.76	0.34	0.120	0.388	0.13	–1.36, 1.62	1.000	
**Alcohol**											0.004
Yes	5.67 ± 2.93	6.58 ± 3.34	–0.91	–1.34	–0.48	**<0.001**	**<0.001**	–0.46	–1.47, 0.55	0.012	
No	4.75 ± 2.62	5.64 ± 3.4	–0.89	–1.96	0.18	0.101	0.175	Ref			
**Smoker**											0.070
Yes	6.55 ± 2.26	8.90 ± 3.66	–2.35	–3.95	–0.75	**0.006**	**0.013**	**–2.29**	**–3.85, –0.73**	**0.001**	
No	5.28 ± 2.74	6.07 ± 3.13	–0.79	–1.20	–0.38	**<0.001**	**0.001**	**Ref**			
**Anxiety/depression**											0.072
Yes	6.69 ± 3.40	7.85 ± 3.13	–1.17	–2.06	–0.27	**0.012**	**0.011**	**–1.74**	**–2.63, –0.85**	**<0.001**	
No	5.14 ± 2.63	5.96 ± 3.32	–0.82	–1.27	–0.37	**<0.001**	**0.004**	**Ref**			
**Mediterranean Diet**											0.012
Yes	5.36 ± 3.07	6.25 ± 3.12	–0.89	–1.62	–0.16	**0.017**	**0.008**	0.65	–1.48, 0.19	0.129	
No	5.57 ± 2.81	6.49 ± 3.48	–0.92	–1.40	–0.43	**<0.001**	**0.003**	Ref			
**Follows Physical Activity Recommendations**											0.000
Yes	5.43 ± 2.93	6.34 ± 3.12	–0.91	–1.43	–0.40	**<0.001**	**0.001**	0.078	–0.85, 0.69	0.842	
No	5.57 ± 2.87	6.47 ± 3.56	–0.91	–1.50	–0.31	**<0.001**	**0.013**	Ref			
**Living situation**											0.004
University residence	5.79 ± 2.78	6.21 ± 3.15	–0.41	–1.36	0.54	0.387	0.455	**Ref**			
Shared apartment	5.53 ± 3.16	6.07 ± 2.94	–0.53	–1.11	0.04	0.069	0.087	–0.17	–1.55, 1.21	1.000	
With family	5.33 ± 2.65	6.81 ± 3.66	–1.48	–2.16	–0.80	**<0.001**	**<0.001**	–0.29	–1.42, 0.85	1.000	
Other	5.40 ± 2.70	8.20 ± 6.57	–2.80	–8.17	2.57	0.221	0.285	–0.86	–4.22, 2.74	1.000	

MD, mean difference; SD, standard deviation; Bold, statistically significant differences.
